# Rapid Monitoring of Vancomycin Concentration in Serum Using Europium (III) Chelate Nanoparticle-Based Lateral Flow Immunoassay

**DOI:** 10.3389/fchem.2021.763686

**Published:** 2021-10-18

**Authors:** Lun Bian, Junyu Liang, Hui Zhao, Ke Ye, Zhaoyue Li, Tiancai Liu, Jie Peng, Yingsong Wu, Guanfeng Lin

**Affiliations:** ^1^ Key Laboratory of Antibody Engineering of Guangdong Higher Education Institutes, Institute of Antibody Engineering, School of Laboratory Medicine and Biotechnology, Southern Medical University, Guangzhou, China; ^2^ Department of Clinical Laboratory, The Third Affiliated Hospital of Guangzhou Medical University, Guangzhou, China; ^3^ Department of Plastic and Aesthetic Surgery, Third Affiliated Hospital, Sun Yat-sen University, Guangzhou, China; ^4^ Department of Infectious Diseases, Nanfang Hospital, Southern Medical University, Guangzhou, China; ^5^ Experimental Center of Teaching and Scientific Research, School of Laboratory Medicine and Biotechnology, Southern Medical University, Guangzhou, China

**Keywords:** vancomycin, lateral flow immunoassay, Eu (III) chelate nanoparticles., therapeutic drug monitoring, point of care test (POCT)

## Abstract

Establishing personalized medication plans for patients to maximize therapeutic efficacy and minimize the toxicity of vancomycin (VAN) requires rapid, simple, and accurate monitoring of VAN concentration in body fluid. In this study, we have developed a simple and rapid analytical method by integrating Eu (III) chelate nanoparticles (CN-EUs) and lateral flow immunoassay (LFIA) to achieve the real-time monitoring of VAN concentration in serum within 15 min. This approach was performed on nitrocellulose (NC) membrane assembled LFIA strips via indirect competitive immunoassay and exhibited a wide linear range of detection (0.1–80 μg*ml^−1^) with a low limit of detection (69.2 ng*ml^−1^). The coefficients of variation (CV) of the intra- and inter-assay in the detection of VAN were 7.12–8.53% and 8.46–11.82%, respectively. The dilution test and specificity indicated this method had a stability that was not affected by the serum matrix and some other antibiotics. Furthermore, the applicability of the proposed method was assessed by comparing the determined results with those measured by LC-MS/MS, showing a satisfactory correlation (*R* ^2^ = 0.9713). The proposed CN-EUs-based LFIA manifested promising analytical performance, which showed potential value in the real-time monitoring of VAN and could help optimize the clinical use of more antibiotics.

## Introduction

Methicillin-resistant staphylococcus aureus (MRSA) is one of the main pathogens of hospital and community infection (Pujol et al., 2021). It has a strong pathogenicity and can cause skin and soft tissue infection, blood infection, and all organs infection (Genardi et al., 2020). In recent years, it has spread around the world at an alarming rate (Yagnik et al., 2021). MRSA has a broad spectrum of drug resistance to frequently-used β-lactam and cephalosporin antibiotics, as well as to aminoglycosides, macrolides, tetracyclines, fluoroquinolones, sulfonamides, and rifampicin with varying degrees, which makes its clinical management very challenging (Dinarvand et al., 2020). Fortunately, MRSA remains susceptible to vancomycin (VAN), one of the strongest antibiotics as yet known, which is recommended intravenously as a first-line treatment for complicated infections caused by MRSA (Holubar et al., 2020). Although VAN has been around for more than half a century, only nine vancomycin-resistant staphylococcus aureus have been found in the world (Jiang et al., 2021). VAN is also a relatively safe choice for severe infections caused by gram-positive microbes, as it works effectively through inhibiting the growth and reproduction of bacteria (Metlay et al., 2019). Applied as the first choice treatment of MRSA (Ragunathan et al., 2018), clinical use of VAN is particularly important, and rational use is highly important but being challenged by several reasons due to its drug toxicity and metabolic features. First, the toxicity of VAN can cause serious adverse reactions, including rash, phlebitis, and ear damage, the most serious of which is kidney injury that needs dynamic therapeutic monitoring during the treatment (Chattaweelarp et al., 2020). Moreover, when the drug concentration cannot reach the ideal range due to individual differences in drug metabolic rate, this results in treatment failure and induces drug resistance (Cong et al., 2020). The above challenges make the therapeutic drug monitoring (TDM) of the blood level of VAN on patients vitally necessary in clinical use (Jeffres, 2017).

Under the development model of precision medicine, new requirements are put forward for TDM. Rapidity, simplicity, and accuracy of drug concentration monitoring is the trend of its clinical application (Liang et al., 2019). The existing analytical methods for detecting VAN concentration monitoring mainly include enzyme-linked immunosorbent assay (ELISA) (Odekerken et al., 2015), high-performance liquid chromatography (HPLC) (Fan et al., 2020), liquid chromatography mass spectroscopy/mass spectroscopy (LC-MS/MS) (Moorthy et al., 2020) alone or combined with capillary electrophoresis (CE) (Sánchez-Hernández et al., 2014), fluorescence spectroscopy, and fluorescence polarization (Dey et al., 2018). Although the accuracy of these existing methods has improved from ELISA to HPLC or LC-MS, there is no substantial development in terms of simplicity and speed. Shortcomings such as high cost, complicated operation, and high technical requirements are still immanent. Most significantly, the large time consumption, caused by sample treatment and detection procedures, leads to the catabolism of VAN in the patient’s body, which makes the results unapplicable as a reference for correcting the dosage of the drug (Klapkova et al., 2020). Hence, most of those methods are inappropriate for popularization for TDM of VAN owing to these disadvantages. In contrast, membrane-based LFIA is an emerging method universally referred to as a point-of-care test that integrates immunoreaction with chromatography and has several advantages, such as simple steps without intricate sample treatment, short assay time, wide detection range, low cost, and high precision and specificity (Chen H. et al., 2020). The most common type of LFIA is a gold nanoparticle-based LFIA which has been widely used in various fields (Liu J. et al., 2020; Di Nardo et al., 2021). However, this traditional LFIA is limited to offering only qualitative or semi-quantitative results (Huang et al., 2016). In recent years, by combining fluorescence-based LFIAs with portable strip readers, LFIAs can satisfy the requirement of semi-quantification detection (Chen Z. et al., 2020; Kim et al., 2021; Wang et al., 2021). The application of lanthanide-doped polystyrene nanomaterials can remedy the defects of conventional fluorescent dyes, which include photobleaching, poor stability, or poor quantum yield, and the advantages of simplicity and rapidity can be retained (Tang et al., 2017). Furthermore, lanthanide chelates are appropriate as immunoassay markers for trace analysis with the advantages of long fluorescence decay time, large Stoke shift between excitation light and emission light, narrow excited fluorescence band, sharp fluorescence emission, and excellent photo-stability, all of which lead to outstanding analysis sensitivity and accuracy (Liu et al., 2021).

In this study, we constructed an LFIA method to detect VAN. Using carboxylate-modified polystyrene CN-EUs as a reporter, residual VAN is detected to enable dosage adjustment based on the real-time monitoring of the VAN metabolism rate in patients. The result of the proposed LFIA was quantified by the ratio of the fluorescence peak heights of the test line and control line (HT/HC ratio). Simultaneously, we evaluated the performance of the CN-EUs-based LFIA, including linearity, reproducibility, analytical sensitivity, and accuracy. In this study, 19 clinical samples were tested using the CN-EUs-based LFIA.

## Results

### Characterization of CN-EUs and Coupled CN-EUs

**FIGURE 1:**
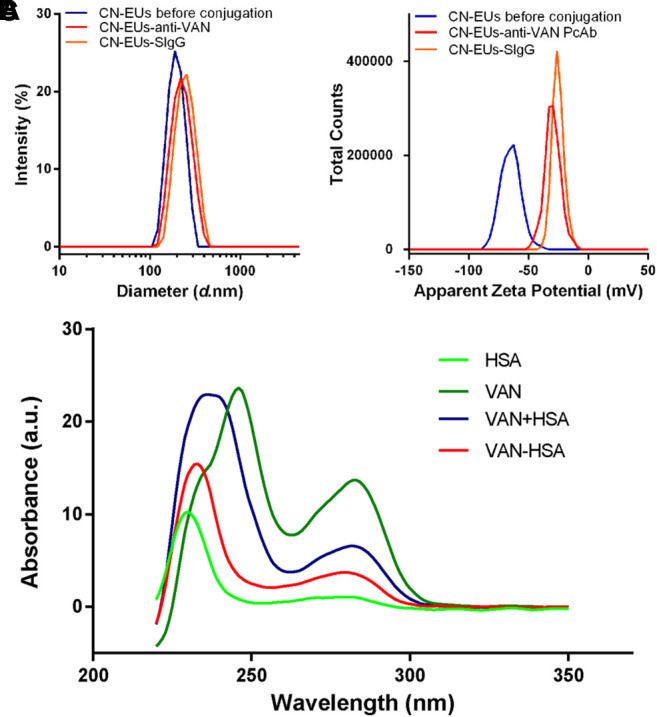
Validation of conjugated nanoparticles and competitive antigen. The **(A)** particle size and **(B)** Zeta potential of CN-EUs. **(C)** The UV-VIS spectra of VAN-HSA (VAN coupled with HSA) and VAN + HSA (mixture of VAN and HSA).

The characterization of CN-EUs and coupled CN-EUs were analyzed using Zetasizer software (version 7.13, Macromedia Inc., San Francisco, CA, United States). The particle size and Zeta potential of CN-EUs are shown in Figures 1A,B. The average particle size of uncoupled CN-EUs and those conjugated with sheep IgG (SIgG) or anti-VAN polyclonal antibodies (PcAbs) were 192.7, 241.1 and 218.8 nm with polymer dispersity index (PDI) of 0.008, 0.024 and 0.025, and the Zeta potential of those were −65.0, −25.4, and −29.7, respectively. There were significant differences in particle size and Zeta potential between conjugated and uncoupled nanoparticles.

### Characterization of the VAN-HSA Conjugate

As shown in Figure 1C, using unconjugated protein as a blank control, the purified VAN conjugated antigen was identified by ultraviolet-visible spectroscopy (UV-VIS). The characteristic absorption peak of VAN is at 280 nm. The absorbance curves of VAN-human albumin (HSA) synthetic and the mixture of VAN and HSA were also showed an absorption peak at 280 nm, which indicated that VAN was linked with the carrier protein successfully.

### Principle of the Method

**FIGURE 2:**
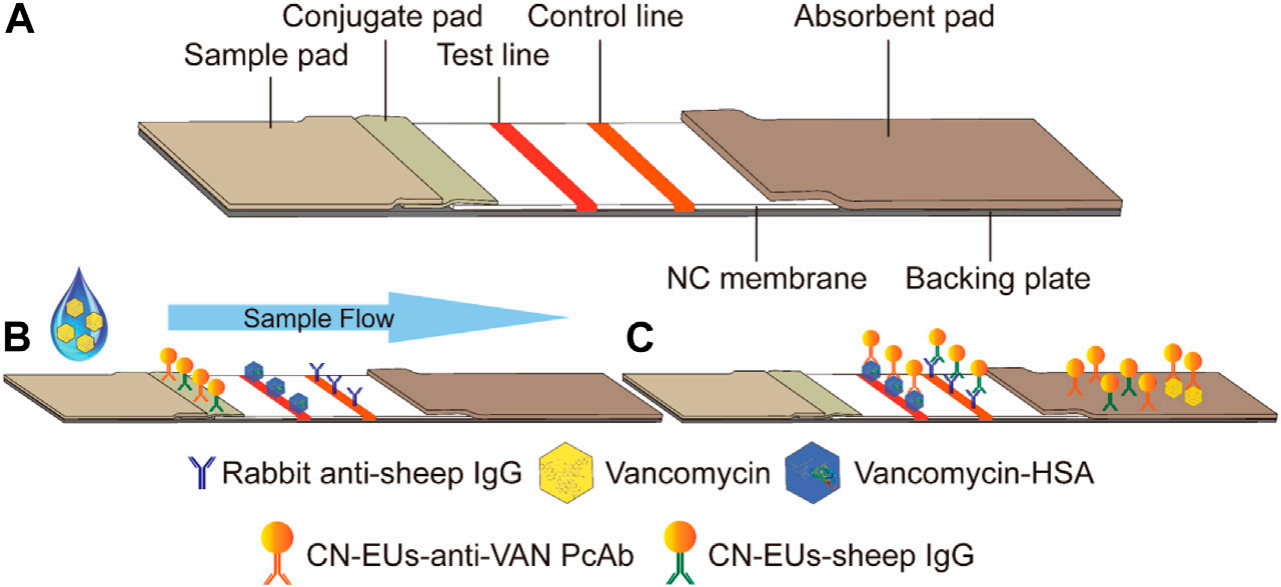
Schematic illustration of the CN-EUs-based LFIA. **(A)** Pattern diagram of CN-EUs-based lateral flow test strip. **(B)** The conjugated VAN-HSA and the anti-SIgG are immobilized on the test line and the control line, respectively. Samples containing VAN are added to the sample pads and migrate along the NC membrane by capillary action, **(C)** and the VAN-HSA competes with the VAN in the sample for binding to the anti-VAN PcAbs.

The test strips are assembled and cut as shown in Figure 2A. The LFIA for testing VAN concentration was performed as a typical competitive time-resolved fluoroimmunoassay, illustrated in Figures 2B,C. When the sample buffer containing VAN was added to the sample pad, the mixture migrated to the conjugated pad by capillarity and partially combined with CN-EUs labeled anti-VAN PcAbs. After the complexes flow reached the NC membrane, the uncombined nanoparticles labeled anti-VAN PcAbs were captured by the test line once they had arrived, while the nanoparticles labeled SIgG were captured by control line, respectively. Subsequently, the superfluous fluorescent nanospheres migrated into the absorption pad. The test strip was then measured using a time-resolved fluorescence (TRF) reader to obtain the peak heights of the test line and the control line. In the competitive assay system, the VAN-HSA coating on the test line competed with the VAN in the sample for binding to the conjugates of CN-EUs with anti-VAN PcAbs, with the result that if there os more VAN in the sample, the lower fluorescent signal intensity appears on the test line. Therefore, an inverse relationship occurred between the fluorescence intensity of the test line and VAN concentration in the sample. On the other hand, as an internal control to confirm that the sample had migrated the lines and reacted correctly, the fluorescence intensity of the control line was almost constant, regardless of VAN concentration in the sample. Conclusively, the ratio of H_T_/H_C_ was used as the assay result, which could counteract the intrinsic heterogeneity of the lateral flow test strip and the sample matrix, showing more reliability and reproducibility than when only H_T_ was used for signal quantitation.

### Optimization of the Immunoreaction Time

**FIGURE 3:**
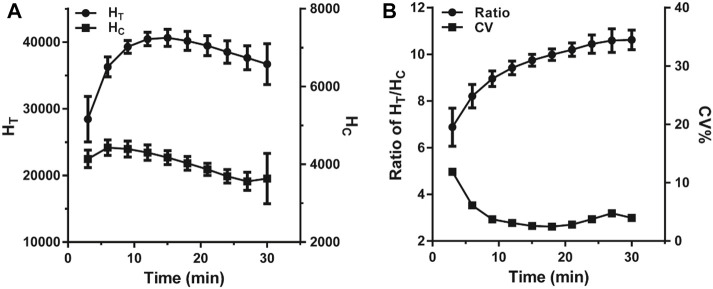
The change trend of fluorescence intensity within 30 min of incubation. **(A)** The change of the fluorescence peak heights. **(B)** The change of the ratio of H_T_/H_C_.

The reaction time is a significant factor that heavily influenced the fluorescence intensity variation in the LFIA. In this case, a VAN standard sample of 0 ng*ml^−1^ was used to evaluate the effect of reaction time by monitoring H_T_, H_C_, and H_T_/H_C_ ratio. As illustrated in Figure 3A, over the range of 3–30 min of incubation, each value was based on ten replicated measurements and assessed per 3 min. The H_T_ and H_C_ sharply rose and reached their peak values in 12 and 6 min, respectively. Subsequently, the H_T_ stood at equilibrium for 3 min and then declined slowly, while the H_C_ fell immediately once reaching its peak. As shown in Figure 3B, the H_T_/H_C_ ratio dropped continually during the reaction, and the minimum standard deviation presented at 15–18 min indicating that the H_T_/H_C_ ratio was most stable. Considering both stability and timeliness, 15 min was selected as the incubation time for further studies.

### Dilution Test

**FIGURE 4:**
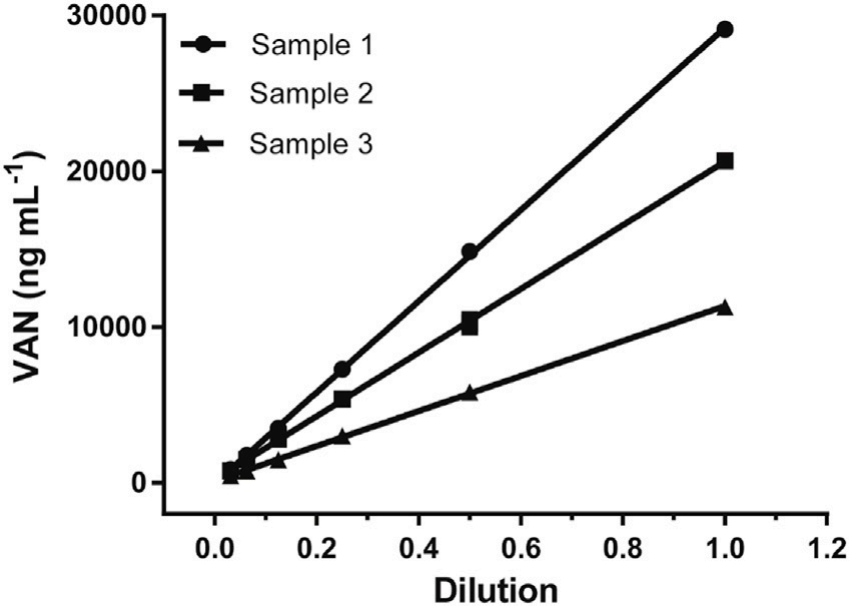
Dilution linearity for VAN based on measurement of three serum samples.

The dilution test was performed to find the reliability of the assay using gradual serial dilution over the range from 1/2 to 1/32 with a sample buffer for three positive sera samples, and then analyzed according to the previous method. As shown in Figure 4, all three sample dilutions exhibited good linearity with an R square above 0.99, indicating little variation in the observed concentrations after correcting for sample dilution. This gives evidence that the proposed LFIA method was suitable for quantitative measurements.

### Linearity and Analytical Sensitivity

**FIGURE 5:**
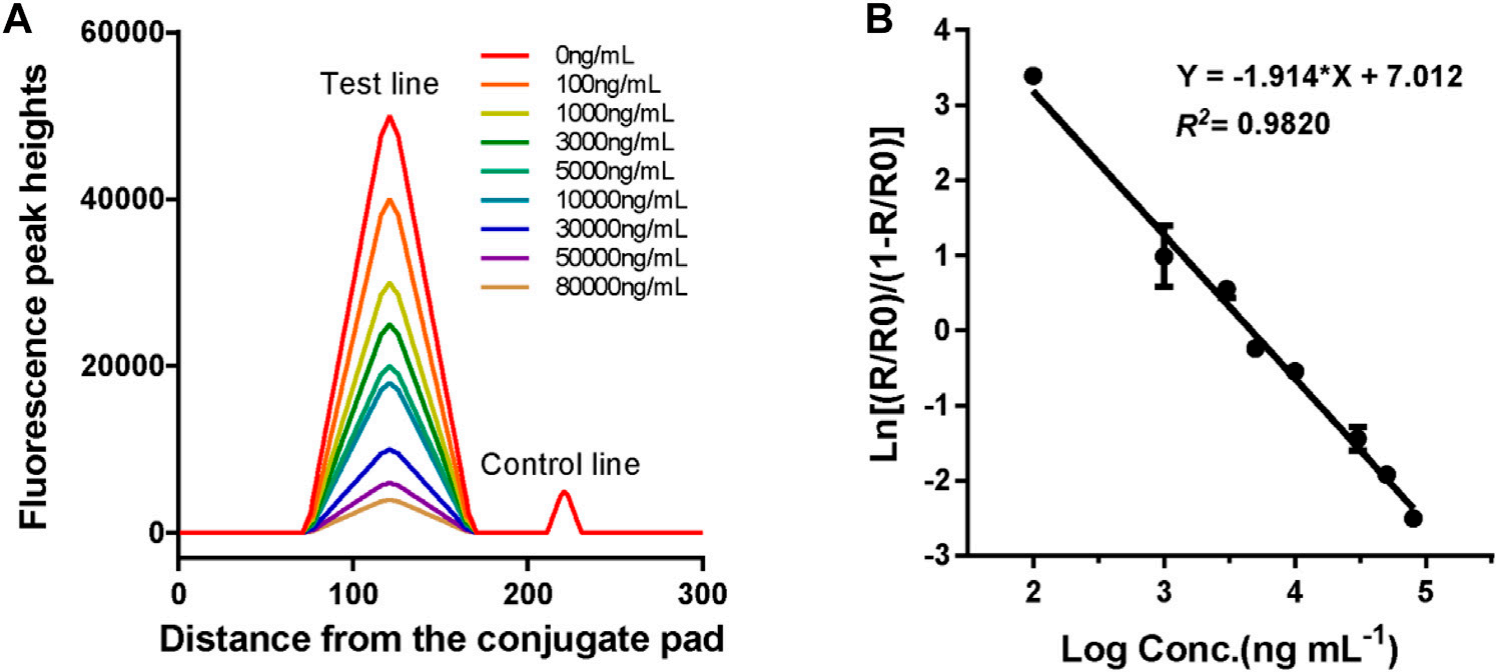
Fluorescence peak readout curve and standard curve of CN-EUs-based LFIA strips for VAN. **(A)** Fluorescence peak heights readout curve for VAN at concentrations of 0–80,000 ng*ml^−1^. **(B)** Standard curve of CN-EUs-based LFIA strips for VAN was obtained for calibration samples from 0 to 80,000 ng*ml^−1^. A logit-log plot was obtained from the computation formula: ln (Y) = −1.914*log(X)+7.012, Y=(Rx/R0)/(1-Rx/R0).

The standard curve for the proposed LFIA was constructed based on the measurement of a series of different concentrations of VAN standards (0, 100, 1,000, 3,000, 5,000, 10,000, 30,000, 50,000, and 80,000 ng*ml^−1^), which were accurately prediluted using a sample buffer. According to the fluorescence intensities recorded using a TRF strip reader as shown in Figure 5A, we obtained the standard curve by plotting the logit (Y) against the logarithm of the VAN concentration as represented by the equation: logit (Y) = 7.012–1.914*log(X), with a reliable coefficient of determination (*R* ^*2*^ = 0.982). The dose-response curve was displayed throughout the whole range of VAN concentration, as shown in Figure 5B. The analytical sensitivity (limit of detection), defined as the concentration corresponding to the mean minus 2*SD (*n* = 15) of the H_T_/H_C_ ratio of the zero standard, was calculated to be 69.2 ng*ml^−1^.

### Reproducibility and Specificity

The reproducibility of the developed LFIA was assessed according to the intra-assay (within a day) and inter-assay (between days) precision. As shown in Table 1, the intra- and inter-assay are 7.12–8.53% and 8.46–11.82%, respectively. All the CVs are around 10%, which indicates an acceptable level of precision for the VAN strip quantification. The specificity was evaluated by the examination of possible interferents at relatively high concentrations using the proposed method, including teicoplanin, penicillin, and cephalosporins. The cross-reactivity was calculated using the formula: cross-reactivity (%) = (measured concentration of VAN)/(expected concentration of interferent). As shown in Table 2, the results demonstrated that the developed polystyrene CN-EUs-based LFIA had a high specificity towards VAN.

**TABLE 1:** Reproducibility test of the present assay.

	Samples	Concentration (ng*ml^−1^)	Mean ± SD (ng*ml^−1^)	CV (%)
Intra-assay (*10)	QC-1	100	100.26 ± 7.14	7.12
	QC-2	1,000	1,047.64 ± 89.41	8.53
	QC-3	10,000	10,416.76 ± 756.46	7.26
Inter-assay (*5)	QC-1	100	99.19 ± 11.73	11.82
	QC-2	1,000	1,020.68 ± 93.77	9.19
	QC-3	10,000	10,207.91 ± 863.86	8.46

**TABLE 2:** Specificity of the proposed method for VAN.

Interferent	Concentration	Measured results (ng*ml^−1^)	Cross-reactivity (%)
Teicoplanin	10,000 (ng*ml^−1^)	26.41	0.2641
Streptomycin	10,000 (ng*ml^−1^)	19.73	0.1973
Cephalosporins	10,000 (ng*ml^−1^)	25.09	0.2509
Penicillin	10,000 (IU*ml^−1^)	12.13	0.1213

### Comparison With LC-MS/MS Assay

**FIGURE 6:**
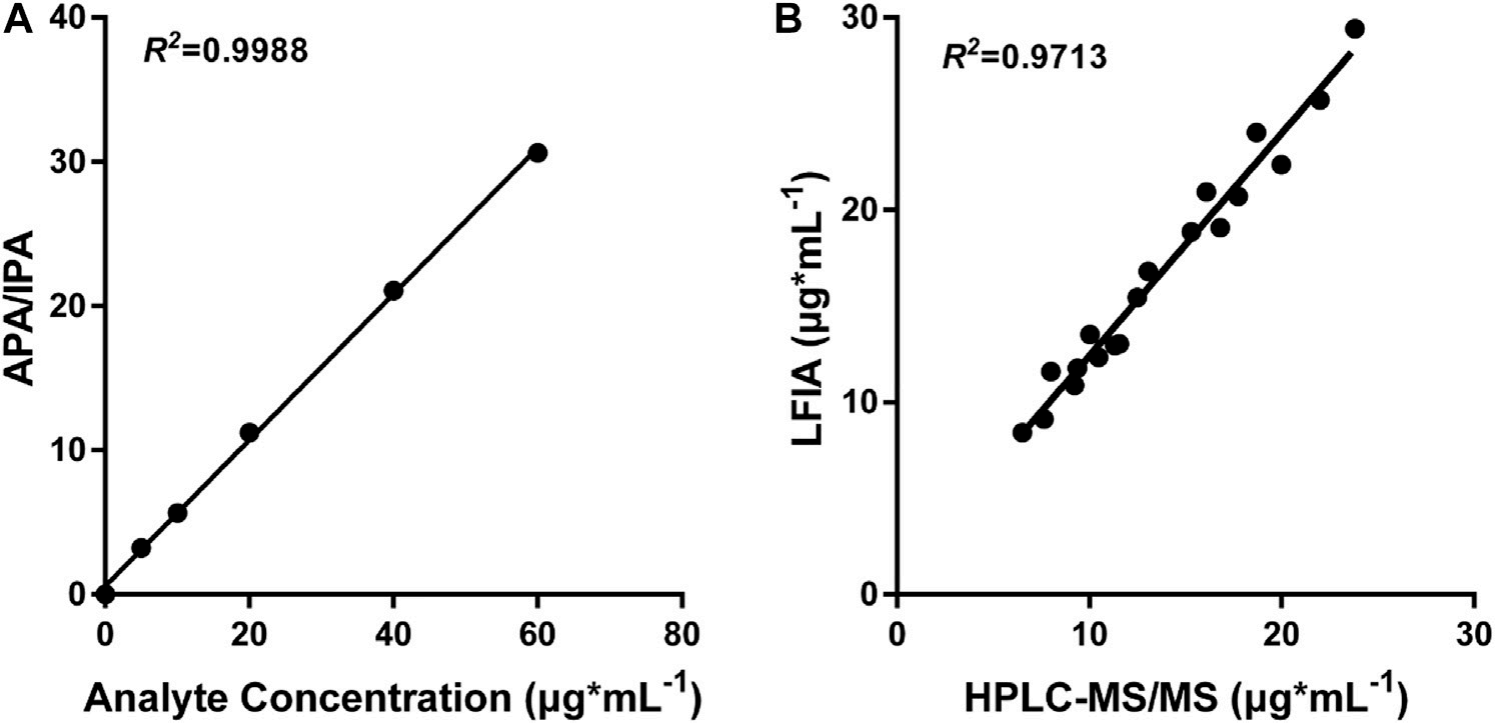
LC-MS/MS analysis and comparison with CN-EUs-based LFIA strips. **(A)** LC-MS/MS calibration curve of VAN according to the following VAN standards: 0, 5, 10, 20, 40, and 60 μg*ml^−1^. APA = analyte peak area, IPA = IS peak area. **(B)** Linear correlation between the proposed CN-EUs-based LFIA and LC-MS/MS (*R* ^*2*^ = 0.9713).

In order to demonstrate the clinical application of the polystyrene CN-EUs-based LFIA system, 19 clinical serum samples were analyzed using the proposed method and LC-MS/MS simultaneously. The LC-MS/MS calibration curve is presented in Figure 6A, with the ratio of analyte peak area/IS peak area as the y-axis and the analyte concentration as the x-axis with an R square of 0.9988. The linear correlation between the two methods is shown in Figure 6B (*R* ^2^ = 0.9713). The results indicate the LFIA has a satisfactory analytical performance and is comparable with the LC-MS/MS method for the determination of VAN concentration in human serum samples.

## Discussion

The LFIA is a very suitable method for the immediate monitoring of drug concentration. An LFIA that qualitatively detects the residual concentration of VAN has been reported before (Kong et al., 2017). However, because its label is gold nanoparticles, it cannot meet the requirements of quantitative detection in TDM. In the past few decades, by labeling lanthanide chelates on the surface of polystyrene nanoparticles, a new immunoassay label with unique fluorescence characteristics, enhanced light stability, and distinguished signal amplification ability has gradually prevailed in immunodiagnostic methods (Finley et al., 2016). Currently, commercial europium (III) [Eu (III)] chelate-dyed nanoparticles-modified carboxylic acid groups can wrap thousands of fluorescent chelates in a single polystyrene shell, thereby providing high lanthanide-specific fluorescence with enhanced labeling strength. Moreover, the structure of carboxylated nanoparticles provides convenience for the application process of chelates. For example, the shell can provide a stable environment to protect it from the interference of the compound in the reaction, and the carboxyl group on the surface can be covalently bound to the protein to enhance the stability of detection (Ham et al., 2015). Our research team has done many related studies and published articles on this type of method, and has relatively mature research experience (Liang et al., 2019; Chen H. et al., 2020). In this paper, we report a simple and rapid point-of-care method integrating CN-EUs with an LFIA for quantitative testing of VAN in serum. This method is performed as a direct competitive immunoassay with an assay time of 15 min, which means a timely result can be used to correct drug dosage. To counteract the aforesaid interferences and heterogeneities, the ratio between the fluorescence intensities of the test line and the control line has been selected to provide a reliable analytical result (Liu Z. et al., 2020). In virtue of its simplicity and outstanding analytical performance, this method was suitable for real-time VAN monitoring to reduce the emergence of kidney injury.

The CN-EUs-based LFIA assay can quantitatively detect VAN concentration with a limit of 69.2 ng*ml^−1^ and a wide linear range of 0.1–80 μg*ml^−1^. The clinical practice guidelines of the Infectious Diseases Society of America recommend that trough serum VAN concentration of patients be controlled at 10–20 μg*ml^−1^ during treatment (Rybak et al., 2020). Our method not only covers this concentration range, but it is also even feasible when the VAN concentration in the patient’s serum reaches the dangerous trough concentration of 30–65 μg*ml^−1^. Compared with the previous ELISA method, with a detection range of 20–5,000 ng*ml^−1^, the developed LFIA has advantages in terms of the maximum measurable concentration of VAN. And only a moderate increase of dilution ratio is required when the sample concentration is > 80 μg*ml^−1^.

In conclusion, we have successfully developed a CN-EUs-based LFIA method that could provide quantitative results of residual VAN in human sera, and has the advantages of convenience, simplicity, rapidity, and low cost, all of which made this proposed strip method appropriate for wide use in primary hospitals. This will improve the efficiency of the real-time monitoring of VAN, to enable the timely adjustment of drug dosage and reduce the occurrence of adverse effects and bacterial drug resistance.

Moreover, due to the different tolerance of patients to the drug, there is the possibility that kidney injury occurs when the trough concentration is lower than 10 μg*ml^−1^, which means that it is not reliable to adjust the dosage for these patients based on the drug concentration alone. Therefore, it is possible for us to develop a novel LFIA method to simultaneously monitor VAN and relevant biomarkers such as neutrophil gelatinase-associated lipocalin (NGAL) (Sinna et al., 2019), cystatin C (Cys-C) (Voulgaris et al., 2019), kidney injury molecule 1 (KIM-1), and β2-microglobulin (β2-MG) (Seibert et al., 2018). This would directly reflect the injury to the patients’ bodies caused by the toxicity of the drug, and make it possible to formulate individualized medication regimens based on the patients’ metabolic levels of drugs. Since a second or even third test line could be coated on an NC membrane, the LFIA is a suitable method to multi-monitor different biomarkers. Based on this research, by coating relevant biomarkers on an NC membrane as a second test line, we would be able to excogitate a simultaneous immunoassay.

## Materials and Methods

### Reagents and Instrumentation

VAN (hydrochloride) (1404-93-9) was obtained from MedChem Express (Monmouth, NJ, United States). Anti-VAN PcAbs (TB2526741) were purchased from Invitrogen (Carlsbad, CA, United States). SIgG Fc fragment (013–0105) and a rabbit-anti-sheep IgG (H&L) (anti-SIgG) (613–4102) were purchased from Rockland Immunochemical Inc. (Limerick, PA, United States). Bovine serum albumin (BSA) was purchased from Roche Diagnostics (Indianapolis, IN, United States). Methanol (MS grade) and CN-EUs were purchased from Thermo Fisher Scientific Inc. (Waltham, MA, United States). Trehalose was obtained from Wako Pure Chemical Industries, Ltd. (Chuo-ku, Osaka, Japan). Sucrose was purchased from Macklin Biochemical Co., Ltd. (Shanghai, China). Sample pads (Ahlstrom 8964) and absorbent pads (H5015) were purchased from Jieyi Biotechnology (Shanghai, China). NC membranes (Hi-Flow Plus HFC13502), conjugate pads (GFCP203000), 0.22 μm syringe filters, and centrifugal filter units with an Ultracel-50 membrane were obtained from Millipore (Bedford, MA, United States). Triton-100, Proclin-300, Polyvinyl alcohol (PVA, average mol wt 30,000–70,000), Polyvinyl pyrrolidone (PVP, average mol wt 10,000), Casein-Na, 4-morpholineethanesulfonic acid (MES), 1-ethyl-3-(3-dimethylaminopropyl) carbodiimide hydrochloride (EDC), N-hydroxysulfosuccinimide (sulfo-NHS), and human albumin (HSA) were purchased from Sigma-Aldrich (St. Louis, MO, United States). All other chemicals were of analytical reagent grade.

Ultrapure water used throughout the study was obtained through a Milli-Q water purification system (Millipore, Bedford, MA, United States). A probe sonicator, UP200S, was purchased from Hielsher (Teltow, Germany). A HulaMixer sample mixer was purchased from Invitrogen (Carlsbad, CA, United States). A zetasizer Nano-ZS90 was obtained from Malvern Panalytical Ltd. (Malvern, Britain). An ultra-microspectrophotometer was purchased from DeNovix Inc. (Wilmington, DE, United States). A BioJet Quant XYZ3060 dispenser was obtained from Biodot Ltd. (Irvine, CA, United States). A draught drying cabinet was purchased from Shelton Manufacturing, Inc. (Cornelius, OR, United States). A strip cutter was purchased from Kinbio Tech. Co., Ltd. (Shanghai, China). An aQcare TRF strip reader was obtained from Medisensor, Inc. (Daegu, Korea).

### Solutions

The buffer solutions used in this study are as follows: sample pad treatment buffer (100 mmol*L^−1^ Na_2_B_4_O_7_.10H_2_O, 0.2% Casein-Na (wt/vol), 1% PVP (wt/vol), 0.1% NaN_3_ (wt/vol) and 6% TritonX-100 (vol/vol)); conjugate pad treatment buffer (50 mmol*L^−1^ Na_2_HPO_4_.12H_2_O, 0.5% BSA (wt/vol), 0.5% PVA (wt/vol) and 1% TritonX-100 (vol/vol), pH 7.4); coating buffer (10 mmol*L^−1^ Na_2_HPO_4_.12H_2_O, 0.3% Trehalose (wt/vol), 0.9%NaCl (wt/vol) and 0.1%NaN_3_ (wt/vol), pH 7.4); activating buffer (25 mmol*L^−1^ MES, pH 6.1); binding buffer (25 mmol*L^−1^ phosphate buffer, pH 7.0); blocking buffer (25 mmol*L^−1^ phosphate buffer, 5% BSA (wt/vol), pH 7.4); washing buffer (25 mmol*L^−1^ Tris, 0.9% NaCl (wt/vol), 0.05% Proclin-300 (vol/vol) and 0.2% Tween-20 (vol/vol), pH7.8); labeling antibody storage buffer (25 mmol*L^−1^ Tris, 5% BSA (wt/vol), 1% Trehalose (wt/vol), 1% sucrose (wt/vol), 0.9% NaCl (wt/vol), 0.05% TWEEN-20 (vol/vol) and 0.05% Proclin-300 (vol/vol), pH 7.2); labeling antibody dilution buffer (20 mmol*L^−1^ Tris, 1% BSA (wt/vol), 5% Trehalose (wt/vol), 20% sucrose (wt/vol) and 0.05% Proclin-300 (vol/vol), pH 9.0); sample buffer (10 mmol*L^−1^ Na_2_HPO_4_.12H_2_O, 1%BSA (wt/vol), 0.9%NaCl (wt/vol), pH 7.4); PBA (10 mmol*L^−1^ Na_2_HPO_4_.12H_2_O, 2 mmol*L^−1^ KH_2_PO_4_.12H_2_O, 0.8%NaCl (wt/vol) and 0.02%KCl (wt/vol), pH 7.4). All solutions were freshly prepared and filtered using 0.22 μm syringe filter before use.

### Processing of Sample Pads and Conjugate pads

The sample pads and conjugate pads were important ingredients of the CN-EUs-based LFIA, and both were made from glass fiber. The sample pads were cut into 300 × 17 mm pieces and saturated with sample pad treatment buffer for 2 h at room temperature. Then the pieces were dried at 37°C for 24 h in a draught drying cabinet and stored in an electronic moisture-proof tank at room temperature until use. The conjugate pads were sliced into 300*10 mm pieces and submerged in conjugate pad treatment buffer for 1.5 h at room temperature. The subsequent processing and storage conditions are the same as the sample pads.

### Preparation and Application of Nanoparticles Coupled With Anti-VAN PcAbs and SIgG

The conjugates of nanoparticles and anti-VAN PcAbs/SIgG were prepared using the method described in our previous works, with only minor changes. Initially, 2 mg of CN-EUs (particle size: 199 nm) were centrifuged at 20,600 g for 15 min at 4°C to remove supernatant stock solution before being activated at room temperature in 500 μl activating buffer including EDC and sulfo-NHS with an ultimate concentration of 1.25 mmol*L^−1^ and 10 mmol*L^−1^, respectively. After 30 min gentle shaking at room temperature, the interactant was centrifuged at 29,700 g for 30 min at 4°C and washed twice with l ml washing buffer each time. The sediment was then resuspended in 400 μl binding buffer by sonication. Subsequently, 25 μg of specific anti-VAN PcAbs/SIgG was purified and concentrated into 100 μl binding buffer by centrifugal filter unit and an Ultracel-50 membrane was added, and then the coupled reaction proceeded for 2 h at room temperature with gentle upside-down mixing by sample mixer. The supernatant containing unlabeled antibodies was removed by centrifugation at 20,600 *g* for 15 min at 4°C and washed twice. Then the mixture was centrifuged again in the previous conditions and the supernatant was aspirated. The precipitate was resuspended with 1 ml blocking buffer and incubated for another 2 h at room temperature with mild shaking. It was then washed three times, and the conjugates that settled at the bottom were blended with labeling antibody storage buffer at a CN-EUs concentration of 2 mg*ml^−1^. After verification of the conjugated CN-EUs, the conjugate solution containing the CN-EUs coupled with anti-VAN PcAbs and SIgG with a final concentration of 0.15 mg*ml^−1^ and 0.03 mg*ml^−1^ was sonicated with a probe at 0.5 cycle and 40% amplitude for 5 min and then distributed on a conjugate pad by the BioJet Quant XYZ-3060 dispenser at a rate of 10 μl*cm^−1^. The pad was dried again and stored in a moisture-proof cabinet at room temperature.

### Preparation of VAN-Conjugate Competitive Antigens

The competitive antigens was designed to use HSA as a carrier protein and were prepared using the following method. First, 10 mg of HSA was dissolved in 500 μl of activating buffer including EDC and sulfo-NHS with a final concentration of 1.25 mmol*ml^−1^ and 10 mmol*ml^−1^, respectively, and uniformly mixed at room temperature for 30 min. The activated mixture was then centrifuged at 9,000 g for 10 min at 4°C by centrifugal filter unit with an Ultracel-50 membrane and washed using a binding buffer three times. After being collected and adjusted to 500 μl with the binding buffer, the supernatant was mixed with 1 mg of VAN dissolved in 500 μl of binding buffer and gently rotated overnight at 4°C. Subsequently, the synthesis was dialyzed against PBS at 4°C for 24 h twice. The competitive antigen, VAN-HSA, was identified by ultraviolet-visible spectroscopy (UV-VIS) and then stored at 4°C until use.

### Preparation of Test Strips

The CN-EUs-based LFIA is composed of five ingredients: sample pad, conjugate pad, absorbent pad, NC membrane, and backing plate. Initially, after being equilibrated to room temperature, the competitive antigen and anti-SIgG were concentrated by centrifugation for a final concentration of 3 mg*ml^−1^ and 1 mg*ml^−1^, respectively, and spotted on the NC membrane using the dispenser at a rate of 0.8 μl*cm^−1^, being separated by a distance of 5 mm to serve as the test line and control line respectively. The membrane was then dried overnight at 37°C and stored in a moisture-proof cabinet. An absorption pad was cut into 300*22 mm strips without any other treatment. All four components were assembled on a 300*60 mm backing plate, so that the components overlapped sequentially to ensure a direct flow from the sample pad to the absorbent pad by capillarity. The whole plate was then cut into 3 mm wide strips using a strip cutter, as shown in Figure 2A. Each strip was packaged into a shell with a circular sample pad well and a rectangular viewing area marked with the test line and control line. Finally, the wrapped strips were sealed in ziplock plastic bags with desiccant and stored at room temperature for future use.

### Standard, Quality Control, and Clinical Serum Samples

The VAN was dissolved in ultrapure water at a concentration of 100 μg*ml^−1^ to obtain a working solution. The working solution was then diluted with a sample buffer to concentrations of 0, 100, 1,000, 3,000, 5,000, 10,000, 30,000, 50,000, and 80,000 ng*ml^−1^ as VAN standards, and diluted with a blank serum to give final concentrations of 100, 1,000, and 10,000 ng*ml^−1^ as quality control samples (QCs) of low, medium and high concentrations, respectively. A total of 19 patients’ serum samples was provided by Nan-fang Hospital (Guangdong, China). All samples were stored at −80°C after immediately after acquisition and unfrozen at 4°C only before use. The study was approved by the Ethical Committee of the Science and Technology Department of the Southern Medical University.

### Fluorescence Lateral Flow Immunoassay Procedure

The proposed LFIA for assessing VAN was performed as a competitive time-resolved fluoroimmunoassay. First, 5 μl of the standards or samples was added to 300 μl of the sample buffer and blended thoroughly. Subsequently, 60 μl of the mixture was loaded onto the sample pad well and migrated towards the absorption pad by capillarity as shown in Figure 2B. The shell containing the test strip was then inserted into an aQcare TRF strip reader after 15 min of reaction and the fluorescence value at 613 nm wavelength of each line and the H_T_/H_C_ ratio was measured under the excitation light of 333 nm wavelength. The fluorescence lateral flow procedure and performance measurement results are shown in Figures 2C, 5A.

### Validation of the Proposed LFIA

The mean and standard deviations (SD) of the fluorescence assay were measured at the H_T_/H_C_ ratio of zero standard on the response curve for 15 duplicates. Sensitivity was calculated as the concentration that corresponds to the value of mean minus double SD. The intra- and inter-assay precisions were obtained from analyzing three QC samples prepared using a negative serum with concentrations of 100, 1,000, and 10,000 ng*ml^−1^, respectively. The QCs were tested ten times per day for intra-assay precision and five duplications for three sequential days for inter-assay.

### LC-MS/MS Procedure

The LC-MS/MS method was based on several previous reports with only minor adjustments, using tobramycin as an internal standard (IS) (Brozmanová et al., 2017). The VAN was dissolved and diluted using methanol for final concentrations of 0, 5, 10, 20, and 60 μg*ml^−1^ as a calibration solution. Each calibration was spiked with IS to get a final concentration of 10 μg*ml^−1^. Subsequently, 50 μl of calibration solution was aspirated and mixed with 50 μl methanol, 60 μl 33% trichloroacetic acid, 200 μl H_2_O, 50 μl acetonitrile, and 50 μl 0.5 mol*L^−1^ NH_4_OH. After gentle oscillation blending, the mixture was centrifuged at 18,000 g for 10 min at 4°C. Then 50 μl supernatant was combined with 25 μl acetonitrile and analyzed on API 3200 triple quadrupole tandem mass spectrometers. The results were analyzed with ABI Analyst Software.

### Statistical Analysis

The VAN dose-response curve was obtained by plotting the logit-log against the logarithm of the VAN concentration (X) using GraphPad Prism 6. The H_T_/H_C_ ratios of the zero VAN standard and the other VAN standards were defined as R_0_ and R_x_, respectively. A logit-log plot was acquired from the computational formula: ln(Y) = ln [(R_x_/R_0_)/(1-R_x_/R_0_)] and the fitting of the line of best fit was ln(Y) = A+ B * log(X). The data analysis was accomplished using SPSS 23.0 (SPSS Inc., Chicago, IL, United States). *p* < 0.05 was considered statistically significant.

## Data Availability

The original contributions presented in the study are included in the article/supplementary material, further inquiries can be directed to the corresponding authors.

## Abbreviations

VAN, vancomycin; LFIA, lateral flow immunoassay; CV, coefficient of variation; MRSA, methicillin-resistant staphylococcus aureus; CN-EUs, Eu (III) chelate nanoparticles; PcAbs, polyclonal antibodies
